# Zika virus evades interferon-mediated antiviral response through the co-operation of multiple nonstructural proteins *in vitro*

**DOI:** 10.1038/celldisc.2017.6

**Published:** 2017-03-21

**Authors:** Yaoxing Wu, Qingxiang Liu, Jie Zhou, Weihong Xie, Cheng Chen, Zefang Wang, Haitao Yang, Jun Cui

**Affiliations:** 1 Key Laboratory of Gene Engineering of the Ministry of Education, State Key Laboratory of Biocontrol, School of Life Sciences, Guangzhou, China; 2 School of Life Sciences, Tianjin University, Tianjin, China; 3 Tianjin International Joint Academy of Biotechnology and Medicine, Tianjin, China; 4 Collaborative Innovation Center of Cancer Medicine, Sun Yat-sen University, Guangzhou, China

**Keywords:** antiviral immunity, nonstructural proteins, type I interferon signaling, Zika virus

## Abstract

Type I interferon (IFN) serves as the first line of defense against invading pathogens. Inhibition of IFN-triggered signaling cascade by Zika virus (ZIKV) plays a critical role for ZIKV to evade antiviral responses from host cells. Here we demonstrate that ZIKV nonstructural proteins NS1, NS4B and NS2B3 inhibit the induction of IFN and downstream IFN-stimulated genes through diverse strategies. NS1 and NS4B of ZIKV inhibit IFNβ signaling at TANK-binding kinase 1 level, whereas NS2B-NS3 of ZIKV impairs JAK–STAT signaling pathway by degrading Jak1 and reduces virus-induced apoptotic cell death. Furthermore, co-operation of NS1, NS4B and NS2B3 further enhances viral infection by blocking IFN-induced autophagic degradation of NS2B3. Hence, our study reveals a novel antagonistic system employing multiple ZIKV nonstructural proteins in restricting the innate antiviral responses.

## Introduction

Zika virus (ZIKV), an arbovirus belonging to the *Flaviviridae* family, was initially isolated from a rhesus monkey in Uganda as early as 1947 [1]. ZIKV infection was previously thought to be asymptomatic, or to cause a mild, flu-like illness [2]. However, ZIKV caught people’s attention during the current wide-spread epidemic in the south Pacific, and south and central America with millions of people infected [3, 4]. Mounting evidence has linked ZIKV infection to serious complications, such as the neurological autoimmune disorder Guillain–Barré syndrome and microcephaly in the newborns of mothers infected during pregnancy [5–7]. In particular, ZIKV was observed to cross the placental barrier to infect human embryonic cortical neural progenitor cells, thus disrupting brain development [8, 9]. Recently, the ZIKV infection has been shown to damage the mouse testes, posing a potential threat to mammalian reproductive system [10]. However, there are no clinically proved vaccines or drugs available to treat ZIKV infection. Hence, there is a pressing need for comprehensive understanding of the molecular pathogenesis of ZIKV to aid in the development of effective vaccines and antiviral therapies.

As an early response to viral infection, type I interferon (IFN) produced by mammalian cells exerts antiviral activity [11]. Activation of IFN signaling initiates from the recognition of pathogen-associated molecular patterns by different pattern recognition receptors [12]. RIG-I-like receptors (RLRs), which recognize cytosolic viral RNA [13], have emerged as critical sensors for RNA viruses, including ZIKV [14]. The binding of viral RNA triggers conformation changes of RIG-I that exposes its CARD domains for subsequent interaction with mitochondrial antiviral signaling adapter (MAVS) [15]. This in turn allows the recruitment of TANK-binding kinase 1 (TBK1) to the signalosome and phosphorylation of interferon regulatory factor 3 (IRF3) to initiate type I IFN transcriptions [16]. After binding to two IFN receptor subunits (IFNAR1 and IFNAR2), type I IFN transduces the signal through the Janus kinases (Jak1 and Tyk2) and signal transducers of transcription (STAT1 and STAT2), leading to the induction of various IFN-stimulated genes (ISGs), which establishes the antiviral state of the cells [11].

Recent studies have indicated that several ISGs, such as IFITM1 and IFITM3, can inhibit ZIKV replication [17]. As viruses have co-evolved with hosts, they have gained multiple mechanisms to evade and antagonize host immune responses [18]. The single-stranded positive RNA genome of flaviviruses encodes a polyprotein, which can be processed into three structural (C, prM and E) and seven nonstructural (NS1, NS2A, NS2B, NS3, NS4A, NS4B and NS5) proteins [19]. The nonstructural proteins of flaviviruses have been implicated to be engaged in immune pathogenesis and antagonism [14, 20]. Particularly, the NS5 of ZIKV has recently been reported to target STAT2 for degradation, thus inhibiting type I IFN signaling [21, 22]. However, the roles of the orchestration of ZIKV nonstructural proteins in repressing antiviral immunity are still largely unknown. Here we report that NS1 and NS4B proteins of ZIKV could inhibit type I IFN production at TBK1 level, whereas NS2B-NS3 (NS2B3 for short) inhibited the JAK–STAT signaling downstream of type I IFN by promoting the degradation of Jak1. Meanwhile, NS2B3 also functioned as an inhibitor of virus-induced apoptosis, which may further help virus replication. Interestingly, we found that, although type I IFN restricted ZIKV replication by promoting the autophagic degradation of NS2B3, NS1 and NS4B inhibited type I IFN production to stabilize NS2B3 during viral infection. Taken together, our findings reveal a synergistic effect of NS1, NS4B and NS2B3 of ZIKV in restricting cellular antiviral responses at multiple levels.

## Results

### ZIKV nonstructural proteins NS1 and NS4B inhibit type I IFN activation

**Figure 1:**
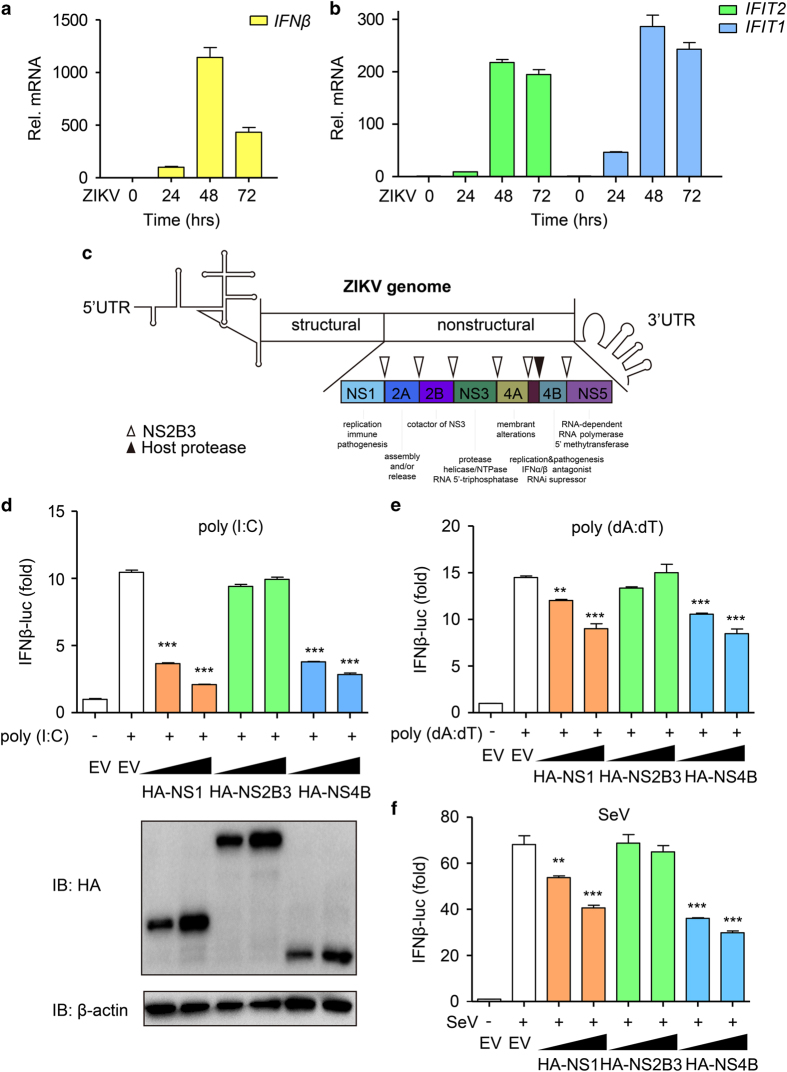
NS1 and NS4B of ZIKV inhibit RLR-induced IFNβ activation. (**a**, **b**) Quantitative PCR with reverse transcription analysis of *IFNβ* mRNA (**a**), *IFIT1* and *IFIT2* mRNA (**b**) in A549 cells infected with ZIKV (MOI=10) for the indicated time points. (**c**) The schematic map of ZIKV genome. (**d**–**f**) Luciferase activity in 293T cells transfected with IFNβ luciferase reporter, together with empty vector (EV) or increasing amounts of HA-NS1, NS2B3 or NS4B of ZIKV. Then, the cells were transfected with cytoplasmic poly(I:C) (5 μg ml^−1^) (**d**), poly (dA:dT) (2.5 μg ml^−1^) (**e**) or infected with SeV (MOI=0.1) (**f**) for 24 h. Data in **a**, **b** and **d**–**f** are expressed as means±s.d. of at least three independent experiments. **P<0.01, ***P<0.001.

Although neural cells are primary hosts for ZIKV, it has been proved that other cell types, such as HeLa and A549 cells, are also permissive for ZIKV [17, 23]. To investigate the role of type I IFN during ZIKV infection, A549 cells were infected with ZIKV (UG MR766 strain). The *IFNβ* and its downstream *ISG*s, including *IFIT1* and *IFIT2*, were induced by ZIKV infection (Figure 1a and b). We also assessed the dynamic change of type I IFN in other cell lines, such as HeLa and brain cancer cell lines. The expression of *IFNβ* and ISGs showed the similar pattern as in A549 cells (Supplementary Figure S1A). As we found that the *IFNβ* expression induced by ZIKV was relatively low compared with other viruses (Supplementary Figure S1B), we hypothesized that ZIKV would inhibit type I IFN production via unidentified mechanisms. There are seven nonstructural proteins (NS1, NS2A, NS2B, NS3, NS4A, NS4B and NS5) of ZIKV (Figure 1c). To investigate the roles of nonstructural proteins in IFN antiviral response, we examined the function of these nonstructural proteins in type I IFN signaling. NS1, NS2B3, NS4B and NS5 significantly inhibited *IFIT2* expression after Sendai virus (SeV) infection (Supplementary Figure S1C). NS5 of ZIKV has been identified to inhibit type I IFN signaling by degrading STAT2 [21, 22]. Besides NS5, NS1 and NS4B of flavivirus are related to immune pathogenesis and NS3 is a protease crucial for the cleavage of viral proteins with the help of NS2B [19]. So we sought to identify the roles of NS1, NS2B3 and NS4B in the regulation of type I IFN signaling pathway. Overexpression of NS1 or NS4B alone but not NS2B3 inhibited cytoplasmic poly (I:C), poly (dA:dT) as well as SeV-induced IFNβ reporter activation in 293T cells (Figure 1d–f). These results suggest that NS1 and NS4B of ZIKV may evade antiviral immunity by directly blocking type I IFN production.

### NS1 and NS4B of ZIKV suppress type I IFN signaling by targeting TBK1

**Figure 2:**
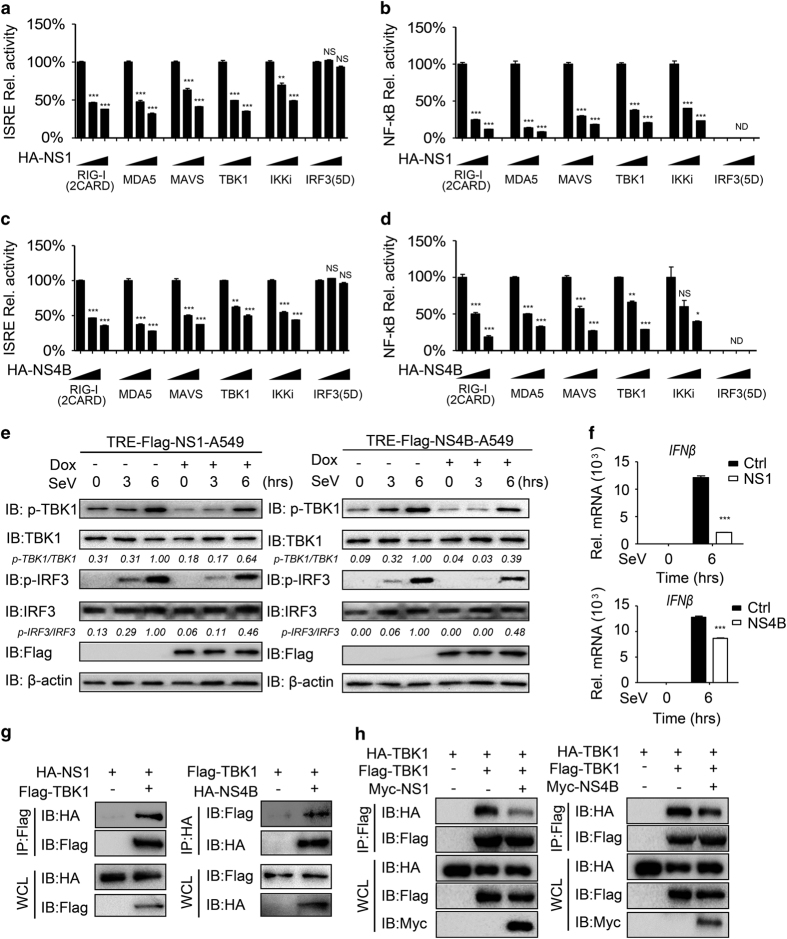
NS1 and NS4B inhibit type I IFN signaling by targeting TBK1. (**a**, **b**) Luciferase activity in 293T cells transfected with an ISRE luciferase reporter (**a**) or NF-κB luciferase reporter (**b**), together with vectors for RIG-I (2CARD), MDA5, TBK1, IKKi and IRF3 (5D), along with empty vector or with expression vectors for NS1. (**c**, **d**) Luciferase activity in 293T cells transfected with an ISRE luciferase reporter (**c**) or NF-κB luciferase reporter (**d**), together with vectors for RIG-I (2CARD), MDA5, TBK1, IKKi and IRF3 (5D), along with empty vector or with expression vectors for NS4B. (**e**) Immunoassay of extracts of NS1- or NS4B-inducible A549 cells were treated with doxycycline (Dox; 200 ng ml^−1^) for 24 h, followed by SeV (MOI=0.1) for the indicated time points. (**f**) Quantitative PCR with reverse transcription analysis of *IFNβ* mRNA in NS1- or NS4B-inducible A549 cells pretreated with Dox (200 ng ml^−1^), followed by SeV (MOI=0.1) infection for 4 h. (**g**) Co-immunoprecipitation and immunoassay of extracts of 293T cells transfected with Flag-TBK1 and HA-NS1 (left) or HA-NS4B (right). (**h**) Co-immunoprecipitation and immunoassay of extracts of 293T cells transfected with Flag-TBK1, HA-TBK1 and Myc-NS1 or NS4B. Data in **a**–**d** and **f** are expressed as means±s.d. of at least three independent experiments. *P<0.05, **P<0.01, ***P<0.001. ND, determined; NS, not significant.

We then checked how NS1 and NS4B affect type I IFN signaling, and found that both NS1 and NS4B suppressed ISRE-luc and NF-κB-luc activities induced by the two CARD domains of RIG-I (2CARD), MDA5, MAVS, TBK1 and IKKi, but not the active form of IRF3 (IRF3(5D); Figure 2a–d). To further determine whether NS1 and NS4B negatively regulate type I IFN signaling, we established a doxycycline-inducible NS1 or NS4B A549 cell lines using the TetON system. Overexpression of NS1 or NS4B restrained the phosphorylation of TBK1 and IRF3 after SeV infection (Figure 2e). The phosphorylation of p65 and degradation of IκBα were also inhibited by overexpressing NS1 and NS4B (Supplementary Figure S2A and B). Furthermore, the expressions of *IFNβ* and *ISG*s after SeV infection were also inhibited by NS1 and NS4B as expected (Figure 2f; Supplementary Figure S2C and D). We next sought to further investigate how NS1 and NS4B inhibit TBK1 phosphorylation. Co-immunoprecipitation analysis indicated that NS1 and NS4B interacted with TBK1 and blocked TBK1 oligomerization (Figure 2g and h). These results suggest that NS1 and NS4B of ZIKV are inhibitors of RLR-induced IFNβ production by targeting TBK1.

### NS2B3 impairs JAK–STAT signaling pathway by promoting the degradation of Jak1

**Figure 3:**
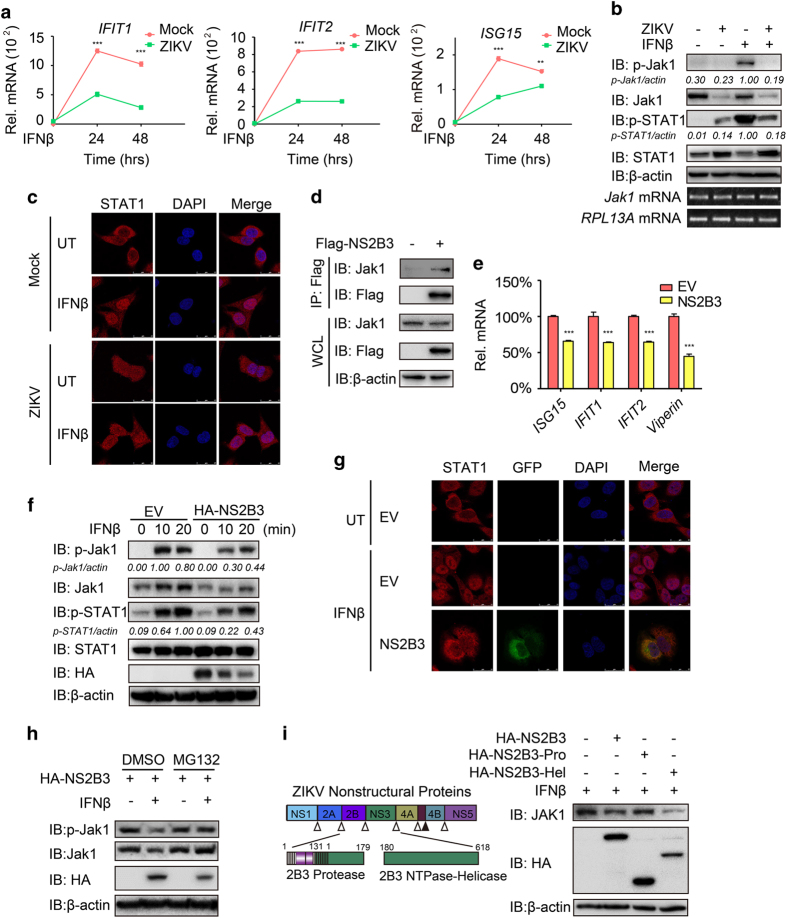
NS2B3 of ZIKV suppresses activation of JAK–STAT signaling by degrading Jak1. (**a**) Quantitative PCR with reverse transcription (qRT-PCR) analysis of *IFIT1*, *IFIT2* and *ISG15* mRNA in A549 cells infected with ZIKV (MOI=10) for 24 h, followed by IFNβ treatment for the indicated time points. (**b**) Immunoassay of extracts of A549 cells treated with or without IFNβ (1 000 U ml^−1^) along with or without ZIKV infection. Below, RT-PCR analysis of *Jak1* mRNA; *RPL13A* mRNA serves as a loading control. (**c**) Confocal microscopy analysis of STAT1 localization in HeLa cells left untreated (UT) or treated with IFNβ after ZIKV pre-infection for 48 h. (**d**) Co-immunoprecipitation and immunoassay of extracts of 293T cells transfected with Flag-NS2B3. (**e**) qRT-PCR analysis of *ISG15*, *IFIT1* and *IFIT2* mRNA in 293T cells transfected with EV or NS2B3 followed by IFNβ treatment (1 000 U ml^−1^) for 3 h. (**f**) Immunoassay of extracts of 293T cells transfected with empty vector (EV) or HA-NS2B3 followed by IFNβ treatment (1 000 U ml^−1^) for indicated time points. (**g**) Confocal microscopy analysis of STAT1 localization in HeLa cells transfected with EV or GFP-NS2B3 followed by IFNβ treatment (1 000 U ml^−1^) for 30 min or left untreated (UT). (**h**) Immunoassay of extracts of 293T cells transfected with HA-NS2B3 and treated with dimethylsulfoxide (DMSO) or MG132 (10 μm) for 4 h. (**i**) Schematic map of constructs of NS2B3 Protease and NTPase-Helicase domains (left). Immunoassay of extracts of 293T cells transfected with HA-NS2B3 (FL), HA-NS2B3 (Pro) or HA-NS2B3 (Hel; right). Data in **a**, **e** are expressed as means±s.d. of at least three independent experiments. **P<0.01, ***P<0.001.

Besides antagonizing the production of IFNβ, several flaviviruses were also reported to prevent the induction of antiviral ISGs by targeting the JAK–STAT signaling pathway [24–26]. We next evaluated ZIKV’s effects on IFNAR signaling pathway. As expected, the replication of ZIKV markedly inhibited the mRNA abundance of IFN-stimulated cytokines including *ISG15*, *IFIT1* and *IFIT2* in response to IFN treatment (Figure 3a). We found that ZIKV infection induced weak activation of Jak1 and STAT1, and reduced the phosphorylation of Jak1 and STAT1 after IFNβ stimulation (Figure 3b). In addition, ZIKV infection also reduced IFNβ-induced nuclear translocation of STAT1 (Figure 3c). Interestingly, we found that ZIKV infection only reduced the protein level of Jak1 but not STAT1 (Figure 3b), while it had little effect on the mRNA level of *Jak1* (Figure 3b). Taken together, our results suggest that ZIKV may block IFN-induced signaling cascade by promoting the degradation of Jak1 protein. In addition, during ZIKV infection, accumulation of ZIKV could enhance its ability to restrict the induction of *ISG*s at later stage of viral infection (Supplementary Figure S3A), suggesting the possibility that the enrichment of ZIKV nonstructural proteins interfered with the induction of IFN-mediated antiviral program. Using immunoprecipitation assays, we found that ZIKV NS2B3 can interact with endogenous Jak1 (Figure 3d). Moreover, the expression of ISGs such as *ISG15*, *IFIT1*, *IFIT2* and *Viperin* was decreased in NS2B3 overexpression cells upon the stimulation of IFNβ (Figure 3e). Furthermore, overexpression of NS2B3 markedly reduced the protein levels of Jak1 but not the levels of STAT1, which consequently inhibited the phosphorylation of Jak1 and STAT1 (Figure 3f), as well as the translocation of STAT1 from the cytoplasm to the nucleus after viral infection (Figure 3g; Supplementary Figure S3B). The degradation of Jak1 can be restored by proteasome inhibitor MG132, suggesting that proteasomal degradation pathway is involved in this process (Figure 3h). The helicase domain of NS2B3 was responsible for the degradation of Jak1 as well as the inhibition of ISGs expression induced by IFNβ and SeV (Figure 3i; Supplementary Figure S3C). In general, we concluded that ZIKV was likely to suppress JAK–STAT signaling by degrading Jak1 through NS2B3.

### NS2B3 blocks RLR-triggered apoptotic cell death

**Figure 4:**
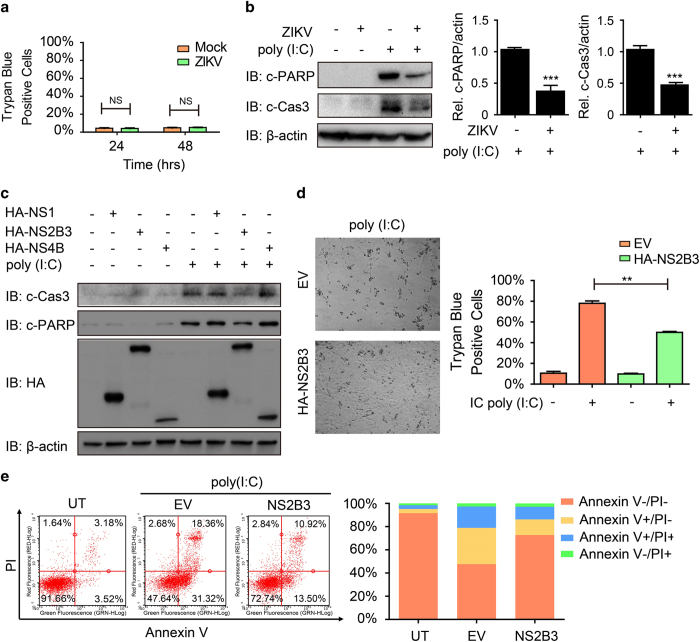
NS2B3 of ZIKV inhibits apoptotic cell death. (**a**) HT1080 cells were infected with ZIKV (MOI=10). Trypan blue-positive dead cells were counted. (**b**) Immunoassay of extracts of HT1080 cells transfected with poly (I:C) (5 μg ml^−1^) for 6 h along with or without ZIKV pre-infection for 48 h. The c-PARP, c-Caspase 3 (c-Cas3) and β-actin were quantified and the relative ratios were calculated. (**c**) Immunoassay of extracts of HT1080 cells transfected with HA-NS1, HA-NS2B3 or HA-NS4B followed by cytoplasmic poly (I:C) (5 μg ml^−1^) treatment for 6 h. (**d**) HT1080 cells transfected with empty vector (EV) or HA-NS2B3 were treated with cytoplasmic poly (I:C) (5 μg ml^−1^) for 12 h. Trypan blue-positive dead cells were counted (right) and photographed (left). (**e**) Fluorescence-activated cell sorting analysis of Annexin V and propidium iodide (PI) staining in HT1080 cells transfected with empty vector (EV) or HA-NS2B3 followed by poly (I:C) (5 μg ml^−1^) treatment for 12 h (left). The ratios of Annexin V-negative and PI-negative (Annexin V−/PI−), Annexin V-positive and PI-negative (Annexin V+/PI−), Annexin V-positive and PI-positive (Annexin V+/PI+), and Annexin V-negative and PI-positive (Annexin V−/PI+) cells were analyzed (right). Data in **a**, **b** and **d** are expressed as means±s.d. of at least two independent experiments. **P<0.01. NS, not significant.

Programmed cell death such as apoptosis is an integral part of host defensing of invading viruses, which helps to restrain the viral proliferation, and is strongly correlated to IFN signaling [27]. The activation of RLRs can also trigger host cell apoptosis [28]. We next wondered whether ZIKV would affect the apoptosis to assist its replication. We observed that trypan blue-positive HT1080 cells (dead cells) did not increase significantly 24 and 48 h post ZIKV infection (Figure 4a), indicating the potential inhibitory functions of ZIKV on apoptotic cell death. ZIKV barely induced the cleavage of caspase 3 and poly ADP ribose polymeraze (PARP) after viral infection (Figure 4b). Furthermore, we found that the cleavage of caspase 3 and PARP induced by cytoplasmic poly (I:C) markedly decreased in the cells, which were pre-infected by ZIKV (Figure 4b). Consistently, cytoplasmic poly (I:C)-induced cell death was inhibited after ZIKV pre-infection (Supplementary Figure S4A). Next, we assessed the functions of NS1, NS2B3 and NS4B in this process. Overexpression of NS2B3, but not NS1 or NS4B, inhibited the IC poly (I:C)-induced cleavage of caspase 3 and PARP (Figure 4c). Consistently, we found that cells overexpressed with NS2B3 were more resistant to cytoplasmic poly (I:C)-induced cell death evidenced by both photographing and trypan blue staining (Figure 4d). This result was further confirmed by Annexin V/propidium iodide staining (Figure 4e). To further investigate whether NS2B3-mediated inhibition of apoptosis is related to NS2B3-mediated degradation of Jak1, we knocked down Jak1 in HT1080 cells and found the cleavage of PARP can still be inhibited by NS2B3 in Jak1 knockdown cells, indicating that NS2B3 inhibits apoptosis in a Jak1-independent manner (Supplementary Figure S4B). Taken together, these data suggest that the NS2B3 of ZIKV attenuates RLR-induced apoptosis.

### Type I IFN restricts the replication of ZIKV and promotes the autophagic degradation of NS2B3

**Figure 5:**
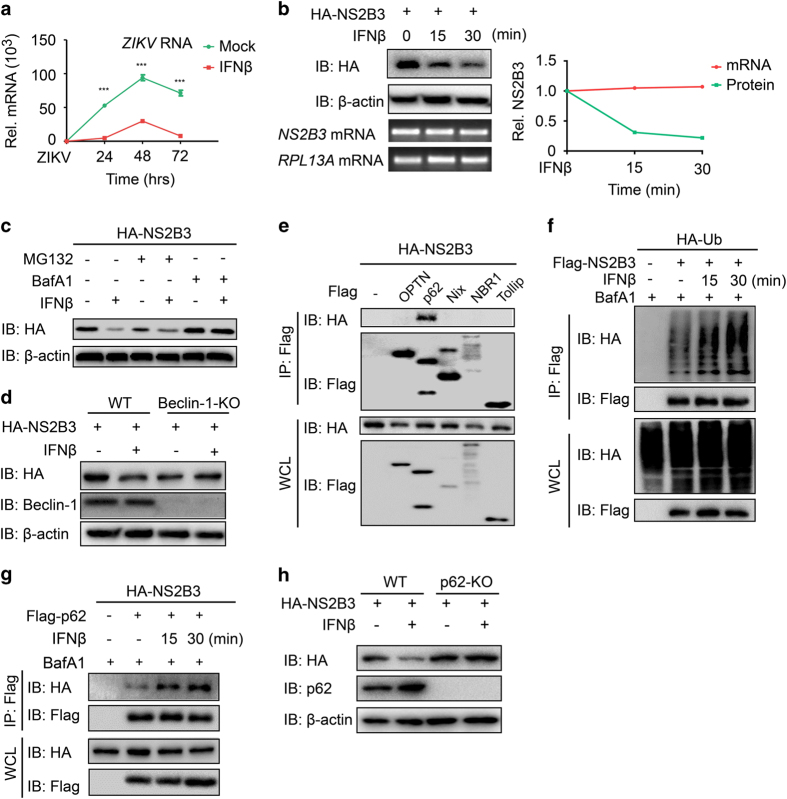
Type I IFN restricts ZIKV replication by promoting the autophagic degradation of NS2B3. (**a**) Quantitative PCR with reverse transcription analysis of *ZIKV genomic RNA* in A549 cells infected with ZIKV (MOI=10) along with or without IFNβ treatment for the indicated time. (**b**) Immunoassay of extracts of 293T cells transfected with HA-NS2B3 followed by IFNβ treatment (1 000 U ml^−1^) for the indicated time. Below, PCR with reverse transcription analysis of *NS2B3* mRNA; *RPL13A* mRNA serves as a loading control. The bands were quantified and the relative NS2B3 level were calculated. (**c**) Immunoassay of extracts of 293T cells treated with or without IFNβ after transfected with HA-NS2B3 and treated with dimethylsulfoxide (DMSO), MG132 (10 μm) or Bafilomycin A1 (BafA1; 20 nm) for 4 h. (**d**) Immunoassay of extracts of wild-type (WT) and Beclin-1-knockout (KO) cells transfected with HA-NS2B3, then treated with or without IFNβ (1 000 U ml^−1^) for 30 min. (**e**) Co-immunoprecipitation and immunoassay of extracts of 293T cells transfected with Flag-OPTN, p62, Nix, NBR1 or Tollip, together with HA-NS2B3. (**f**) Co-immunoprecipitation and immunoassay of extracts of 293T cells transfected with Flag-NS2B3 and HA-Ub, then treated with IFNβ (1 000 U ml^−1^) and BafA1 (20 nm). (**g**) Co-immunoprecipitation and immunoassay of extracts of 293T cells transfected with Flag-p62 and HA-NS2B3, then treated with IFNβ (1 000 U ml^−1^) and BafA1 (20 nm). (**h**) Immunoassay of extracts of wild-type (WT) and p62 KO cells transfected with HA-NS2B3, then treated with or without IFNβ (1 000 U ml^−1^) for 30 min. Data in **a** are expressed as means±s.d. of at least three independent experiments. ***P<0.001.

To better understand the association between ZIKV infection and type I IFN signaling, we set to investigate whether type I IFN restricts ZIKV infection. After IFNβ pretreatment for half an hour, A549 cells were infected with ZIKV. As expected, pretreatment with type I IFN resulted in a marked decrease in the RNA abundance of ZIKV (Figure 5a). Subsequent experiment also showed that IFNβ treatment could promote the degradation of ZIKV NS2B3 but not alter its mRNA level (Figure 5b). As most virus infection including ZIKV resulted in the secretion of IFN, we also determined the expression levels of NS2B3 during SeV infection. Similar with the result of IFNβ treatment, SeV infection can also promote the degradation of NS2B3 (Supplementary Figure S5A). To distinguish the degradation system responsible for the IFN-induced degradation of NS2B3, we treated NS2B3-overexpressing cells with IFN in the presence of proteasome inhibitor MG132 or autophagy inhibitor Bafilomycin A1. We found that IFNβ failed to degrade NS2B3 with the treatment of Bafilomycin A1 rather than MG132 (Figure 5c). Meanwhile, the early-stage autophagy inhibitor 3-methyladenine also blocked the degradation of NS2B3 (Supplementary Figure S5B). Furthermore, IFNβ failed to degrade NS2B3 in Beclin-1 knockout cells, in which the autophagy process is deficient (Figure 5d). Confocal microscopy analysis revealed that the co-localization of NS2B3 and LC3 was enhanced after IFNβ treatment (Supplementary Figure S5C). These results suggest that IFNβ could promote autophagic degradation of NS2B3. Moreover, the degradation of NS2B3 was also in a STAT1-dependent manner (Supplementary Figure S5D). As autophagic cargo receptors are essential for delivering cargoes to the autophagosome for degradation [29], we next investigated which cargo receptors mediate the delivery of NS2B3 to autolysosome. We found that NS2B3 mainly interacted with p62 rather than other receptors (Figure 5e). As ubiquitin chains serve as the major recognition signal for p62-mediated selective autophagic degradation [30], we next confirmed that IFNβ dynamically promoted the ubiquitination of NS2B3 as well as the interaction between NS2B3 and p62 (Figure 5f and g). In addition, IFNβ failed to degrade NS2B3 in p62 knockout cells (Figure 5h). Taken together, these results indicated that IFNβ restricted ZIKV replication and promoted the autophagic degradation of NS2B3 in a p62-dependent manner.

### Co-operation between NS1, NS4B and NS2B3 further attenuates antiviral immunity

**Figure 6:**
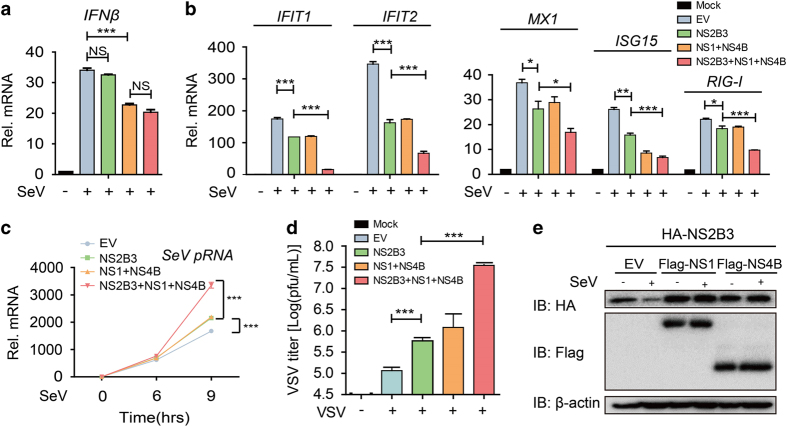
ZIKV nonstructural proteins co-operate to evade IFN restriction. (**a**) Quantitative PCR with reverse transcription (qRT-PCR) analysis of *IFNβ* mRNA in 293T cells transfected with various combinations of plasmid for NS1, NS2B3 and NS4B followed by SeV (MOI=0.1) infection for 9 h. (**b**) qRT-PCR analysis of *IFIT1*,* IFIT2*,* MX1*,* ISG15* and *RIG-I* mRNA in 293T cells transfected with various combinations of plasmid for NS1, NS2B3 and NS4B followed by SeV infection (MOI=0.1) for 9 h. (**c**) qRT-PCR analysis of SeV phosphoprotein RNA (*pRNA*) in 293T cells transfected with various combinations of plasmid for NS1, NS2B3 and NS4B followed by SeV infection (MOI=0.1) for 9 h. (**d**) Plaque titration of VSV in supernatant of 293T cells transfected with various combinations of plasmid for NS1, NS2B3 and NS4B followed by VSV infection (MOI=0.01) for 24 h. (**e**) Immunoassay of extracts of 293T cells transfected with HA-NS2B3, together with Flag-NS1 or NS4B, then infected with or without SeV (MOI=0.1). Data in **a**, **b** and **c** are expressed as means±s.d. of at least three independent experiments. *P<0.05, **P<0.01, ***P<0.001. NS, not significant.

Presuming the negative function of ZIKV NS1 and NS4B against IFN production, we speculated whether NS2B3, NS1 and NS4B might co-operate to antagonize virus-triggered IFN immunity. To confirm this hypothesis, we co-transfected these nonstructural proteins in 293T cells followed with SeV infection. As a result, NS2B3 did not affect the inhibitory function of NS1 and NS4B on *IFNβ* activation (Figure 6a). However, NS1 and NS4B enhanced the inhibitory function of NS2B3 on the induction of several *ISG*s, including *IFIT1*, *IFIT2*, *MX1*, *ISG15* and *RIG-I* after SeV infection (Figure 6b). To clarify the link between attenuated IFN immunity mediated by ZIKV nonstructural proteins and antiviral response, we also monitored mRNA levels of *SeV* to quantify the viral replication. 293T cells co-expressing NS2B3 and NS1 as well as NS4B resulted in higher RNA levels of *SeV* than those only overexpressing NS2B3 or NS1/NS4B alone (Figure 6c). The results were further confirmed by plaque assay after VSV infection. Co-expressing of NS2B3, NS1 and NS4B together resulted in higher VSV titers in supernatants (Figure 6d). These results suggested co-expression of different ZIKV nonstructural proteins rendered the cells more susceptible to viral infection. To determine the mechanism of these findings, we also assessed the SeV-triggered degradation of NS2B3 in NS1 or NS4B co-expressing cells. Interestingly, NS1 or NS4B markedly impaired SeV-induced degradation of NS2B3 (Figure 6e). Collectively, the co-operation between ZIKV NS2B3, NS1 and NS4B further prevents the induction of antiviral ISGs during viral infection, which benefits ZIKV by evading the IFN immune response.

## Discussion

There is no doubt that co-evolution between flaviviruses and their hosts has taken place over a long period of time. Host cells have developed multiple branches of innate immune system to keep the virus invasion and replication under control [14]. Previously, several groups have demonstrated the importance of IFN pathway in restricting different flaviviruses from invasion to replication [14, 31]. Although many studies have reported ISGs such as IFITM family can inhibit the replication of ZIKV [17], little is known about the relationship between host IFN system and ZIKV replication. Here we show that IFNβ restricts replication of ZIKV and promotes autophagic degradation of NS2B3, which broadens our understanding of the host innate immune protective defense against ZIKV. As the ubiquitination of NS2B3 is enhanced by IFN-β treatment and STAT1 is required for the degradation of NS2B3, the potential IFN-inducible E3 ligases might be involved in this process. Many E3 ligases such as tripartite motif (TRIM) proteins family members, including TRIM5α, TRIM23, TRIM25 and TRIM31, can be upregulated by IFN through STAT1 [32, 33]. Most of these TRIM proteins play critical roles in antiviral responses as well as the host restriction on viral replication [33–37]. In this case, further studies are needed to investigate their functions on NS2B3 ubiquitination as well as degradation.

**Figure 7:**
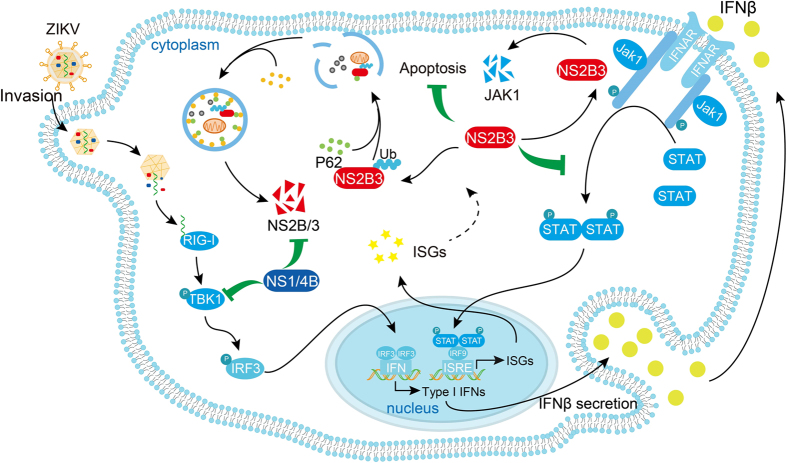
Schematic representation of ZIKV nonstructural proteins antagonize antiviral immunity. ZIKV nonstructural proteins NS1 and NS4B negatively regulate RIG-I-like receptor-induced IFNβ production after viral recognition by inhibiting the phosphorylation of TBK1. Once binding to IFNAR, secreted IFNβ activates Jak1 and STAT1 to initiate ISGs transcription. NS2B3 of ZIKV blocks JAK–STAT signaling by targeting Jak1 for degradation as well as inhibiting virus-induced cell apoptosis. IFNβ can promote the autophagic degradation of NS2B3, whereas NS1 and NS4B inhibit IFN production to stabilize NS2B3, which indicate the co-operation of ZIKV nonstructural proteins in restricting host antiviral immunity.

It has been well documented that different flaviviruses have developed diverse strategies to minimize induction of IFN [19]. Previous studies have identified several strategies for ZIKV to evade the innate immune system. For example, ZIKV NS5 protein could inhibit type I IFN pathway by targeting STAT2 [22]. Here we screened the function of ZIKV nonstructural proteins and showed that they are committed to thwarting the host innate immune response through diverse mechanisms. NS1 and NS4B of ZIKV prevent the activation of RLR-triggered type I IFN induction pathway by targeting TBK1 and subsequently inhibit the synthesis of IFNs. Unlike NS1 and NS4B of ZIKV, NS2B3 does not affect type I IFN production, but impairs JAK–STAT signaling pathway through degrading the tyrosine-protein kinase Jak1 in a proteasome-dependent manner, hence reducing the phosphorylation levels of Jak1 and STAT1. Although the inhibition of ISG expressions may have some feedback effects on IFN production at the later stage of infection *in vivo*, the effects may vary among different cell types, and they may not be strong enough for the feedback effects of JAK–STAT signaling to affect RIG-I signaling in 293T cells, which possibly accounts for the finding that NS2B3 cannot directly inhibit IFNβ activation in our system. NS2B3 also attenuates RLR-induced apoptotic cell death to ensure the complement of viral cell life cycle (Figure 7). It has been noticed that ZIKV could induce apoptosis of neural progenitor cells [38, 39], so we speculated that ZIKV may distinctively regulate apoptosis signaling in different cells upon different stimulations. Further investigations are required to reveal its detail mechanisms in different cell types.

Until now, most studies about flaviruses infection have focused on delineating the function of single nonstructural protein in the antiviral immunity [40]. Here we show that ZIKV nonstructural proteins NS1 and NS4B inhibit the autophagic degradation of NS2B3, thus work collaboratively to minimize the antiviral immunity. Collaborative effort between multiple viral nonstructural proteins should be given more emphasis and attention during elucidating the mechanism of ZIKV infection.

Taken together, we found that multiple nonstructural proteins of ZIKV negatively modulated antiviral response at various levels by inhibiting type I IFN production and the expression of downstream ISGs. Our findings provide evidence to support the potential synergy between ZIKV nonstructural proteins to build up the antagonistic system against innate antiviral immunity, and shed new light on ZIKV immune evading mechanism to suggest novel targets for developing rational vaccine strategies.

## Materials and methods

### Cell culture and reagents

HEK293T, A549, HT1080, HeLa, LN229, U87 and U251 cells were cultured in Dulbecco’s modified Eagle medium (Corning, Manassas, VA, USA) with 10% fetal bovine serum (Genstar, Beijing, China) in a 5% CO_2_ incubator at 37 °C. Recombinant human IFNβ was purchased from Peprotech (Rocky Hill, NJ, USA). Poly (I:C) (LMW) and poly (dA:dT) were purchased from Invivogen (San Diego, CA, USA). Doxycycline (D9891), MG132 (C-2211-5MG), Bafilomycin A1 (H2714) and 3-methyladenine (M9281-100MG) were purchase from Sigma (Shanghai, China).

### Virus infection

Cells were either mock-infected or infected with ZIKV (UG MR766 strain), which was kindly provided by Dr Gucheng Zeng, Sun Yat-sen University. Virus was allowed to adsorb at room temperature for 1 h before incubation at 37 °C for indicated time as previously described [22]. Plaque assays were performed to determine viral titer as previously described [41]. SeV was kindly provided by Dr F Xiao-Feng Qin (Suzhou Institute of Systems Medicine). Cells were infected at different multiplicity of infection (MOI) and time points as indicated.

### Plasmids and antibodies

Plasmids encoding ZIKV NS1, NS2B3 and NS4B were chemically synthesized based on Z1106033 ZIKV strain (NCBI ID: KU312312) and sub-cloned into pcDNA3.1 expression vectors using standard molecular biology techniques. RIG-I (2CARD), MDA5, MAVS, TBK1, IRF3 (5D) and IFNβ, ISRE, NF-κB luciferase reporter plasmids have been previously described [42]. Other plasmids mentioned were acquired by the means of standard PCR techniques. Horseradish peroxidase anti-Flag (M2; A8592) and anti-β-actin (A1978) were purchased from Sigma. Horseradish peroxidase anti-hemagglutinin (HA; 12013819001) was purchased from Roche Applied Science (Shanghai, China). Anti-pTBK1 (5483), anti-TBK1 (3013), anti-pIRF3 (4947s), anti-Jak1 (3344), anti-pJak1 (3331), anti-pSTAT1(9167), anti-C-PARP (5625), anti-C-Caspase 3 (9661), anti-p62 (8025) and anti-Beclin-1 (3738) were acquired from Cell Signaling Technology (Danvers, MA, USA). Anti-IRF3 (sc-9082) and anti-STAT1 (sc-346) were from Santa Cruz Biotechnology (Dallas, TX, USA).

### Immunoprecipitation and immunoblotting

For immunoprecipitation, whole-cell lysates were prepared after transfection, followed incubation overnight with the appropriate anti-Flag beads (Sigma) or anti-HA beads (Sigma). Beads were washed three to five times with low-salt lysis buffer, and immunoprecipitates were eluted with 2× SDS-loading buffer and resolved by SDS-polyacrylamide gel electrophoresis. Proteins were transferred to polyvinylidene fluoride membranes (Bio-Rad, Shanghai, China) and further incubated with appropriate antibodies. LumiGlo Chemiluminescent Substrate System (Millipore, Shanghai, China) was used for protein detection.

### Luciferase reporter assays

293T cells were transfected with a mixture of luciferase reporter (firefly luciferase), pRL-TK (renilla luciferase plasmid), an indicated variety expression plasmid or empty vector (pcDNA3.1) plasmid. Then, the cells were transfected with RIG-I (2CARD), MDA5, MAVS, TBK1 and IRF3 (5D), or stimulated with IC poly (I:C), poly (dA: dT) or infected with SeV. Luciferase activity was measured at 24 h after transfection or infection using a luminometer (Thermo Scientific, Shanghai, China) with a dual-luciferase reporter assay system according to the manufacturer’s instructions (Promega, Beijing, China). Data represent relative firefly luciferase activity, normalized to renilla luciferase activity.

### RNA extraction and quantitative PCR with reverse transcription

Total RNA was extracted using TRIzol reagent (Invitrogen, Shanghai, China) and reverse-transcribed using oligo-dT primers and reverse transcriptase (Vazyme, Nanjing, China). Quantitative real-time PCR was performed using SYBR Green qPCR Mix kit (Genstar) with primers as follows:

*IFNβ*: Forward: 5′- CCTACAAAGAAGCAGCAA-3′

Reverse: 5′- TCCTCAGGGATGTCAAAG-3′

*ISG15*: Forward: 5′- TCCTGGTGAGGAATAACAAGGG-3′

Reverse: 5′- GTCAGCCAGAACAGGTCGTC-3′

*IFIT2*: Forward: 5′- GGAGGGAGAAAACTCCTTGGA-3′

Reverse: 5′- GGCCAGTAGGTTGCACATTGT-3′

*IFIT1*: Forward: 5′- TCAGGTCAAGGATAGTCTGGAG-3′

Reverse: 5′- AGGTTGTGTATTCCCACACTGTA-3′

*MX1*: Forward: 5′- GTTTCCGAAGTGGACATCGCA-3′

Reverse: 5′- CTGCACAGGTTGTTCTCAGC-3′

*VIPERIN*: Forward: 5′- TGGGTGCTTACACCTGCTG-3′

Reverse: 5′- GAAGTGATAGTTGACGCTGGTT-3′

*CCL5*: Forward: 5′- GGCACGCCTCGCTGTCATCCTCA-3′

Reverse: 5′- CTTGATGTGGGCACGGGGCAGTG-3′

*RIG-I*: Forward: 5′- TGTTCTCAGATCCCTTGGATG-3′

Reverse: 5′- CACTGCTCACCAGATTGCAT-3′

SeV pRNA: Forward: 5′- GACGCGAGTTATGTGTTTGC-3′

Reverse: 5′- TTCCACGCTCTCTTGGATCT-3′

*ZIKV genomic RNA*: Forward: 5′- TTGGTCATGATACTGCTGATTGC-3′

Reverse: 5′- CCYTCCACRAAGTCYCTATTGC-3′

*RPL13A*: Forward: 5′- GCCATCGTGGCTAAACAGGTA-3′

Reverse: 5′- GTTGGTGTTCATCCGCTTGC-3′.

### Immunofluorescence

HeLa cells seeded on Glass Bottom culture dishes (Nest Scientific, Jiangsu, China) were fixed with 4% paraformaldehyde for 15 min, and then permeabilized in methyl alcohol for 10 min at −20 °C. After washing with PBS for three times, cells were blocked in 5% fetal goat serum for 1 h, and then incubated with primary antibodies diluted in 10% bull serum albumin overnight. The cells were washed, and followed by a fluorescently labeled secondary antibody (Alexa Fluor 488- and Alexa Fluor 568-conjugated antibodies against mouse, rabbit or goat IgG (Biotium, Hayward, CA, USA)). For viral infection, HeLa cells were either mock-infected or infected with ZIKV (MOI=1) for 48 h following with or without IFNβ for 30 min and fixed with 4% paraformaldehyde, permeablized with methyl alcohol, blocked with 5% fetal goat serum and incubated with the indicated antibodies. The images were photographed by laser scanning confocal microscope (Leica, Wetzlar, Germany).

### Viral plaque titration

293T cells transfected with various combinations of plasmids for NS1, NS2B3 and NS4B were infected by VSV for 18 h. The virus-containing supernatants were collected. Vero cells were infected with VSV supernatants for 1 h at room temperature as described [41]. After washing with PBS, the plate was overlaid with Dulbecco’s modified Eagle medium containing 1% low melting-point agarose and incubate at 37 °C for 24 h before crystal violet staining.

### Statistical analysis

Data are represented as mean±s.d. when indicated, and two-tailed Student’s *t*-test was used for statistical analysis. Differences between groups were considered significant when *P*-value was<0.05.
