# Zika virus evades interferon-mediated antiviral response through the co-operation of multiple nonstructural proteins *in vitro*

**DOI:** 10.1038/celldisc.2017.14

**Published:** 2017-04-11

**Authors:** Yaoxing Wu, Qingxiang Liu, Jie Zhou, Weihong Xie, Cheng Chen, Zefang Wang, Haitao Yang, Jun Cui

**Affiliations:** 1 Key Laboratory of Gene Engineering of the Ministry of Education, State Key Laboratory of Biocontrol, School of Life Sciences,Sun Yat-sen University, Guangzhou, China; 2 School of Life Sciences, Tianjin University, Tianjin, China; 3 Tianjin International Joint Academy of Biotechnology and Medicine, Tianjin, China; 4 Collaborative Innovation Center of Cancer Medicine, Sun Yat-sen University, Guangzhou, China

**Correction to:** *Cell Discovery* (2017) **3**, 17006; doi:10.1038/celldisc.2017.6; published online 21 March 2017

In the initial published version of this article, a mistake was made in the affiliation 1, where ‘Sun Yat-sen University’ should be added after the School of Life Sciences. The corrected affiliation 1 is displayed below. This addition does not affect the results, figures and conclusion of the paper.

^1^Key Laboratory of Gene Engineering of the Ministry of Education, State Key Laboratory of Biocontrol, School of Life Sciences, Sun Yat-sen University, Guangzhou, China;

