# Supplementary material for: Zika virus evades interferon-mediated antiviral response through the co-operation of multiple nonstructural proteins in vitro
Source: Cell Discov. 2017 Mar 21;3:17006–. doi: 10.1038/celldisc.2017.6 (PMC5359216; doi:10.1038/celldisc.2017.6)
Supplement: Supplementary information [file celldisc20176-s1.pdf]

Figure S1

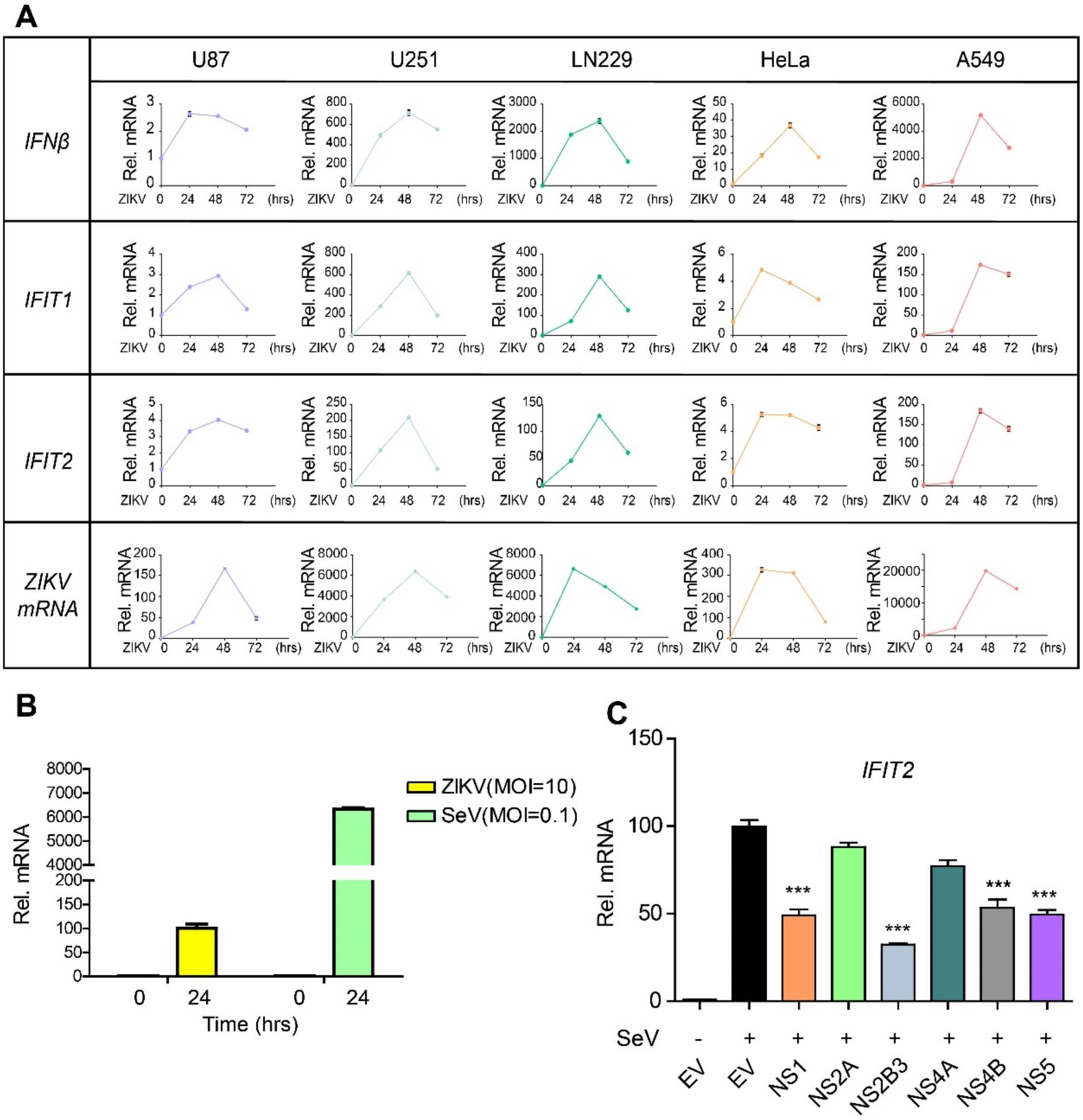

Supplementary Figure S1. ZIKV infection triggers type I IFN antiviral response.

(**A**) qRT-PCR analysis of *IFNβ*, *IFIT1*, *IFIT2* mRNA and *ZIKV genomic RNA* in U87, U251, LN229, HeLa and A549 cells after ZIKV infection (MOI=1) for indicated time periods. (**B**) qRT-PCR analysis of *IFNβ* mRNA in A549 cells infected with ZIKV (MOI=10) or SeV (MOI=0.1) for 24 hrs. (**C**) qRT-PCR analysis of *IFIT2* mRNA in 293T cells transfected with NS1, NS2A, NS2B3, NS4A, NS4B and NS5 followed by SeV infection. Data in **A**, **B** and **C** are expressed as means ± SD of at least three independent experiments. *** p<0.001.

Figure S2

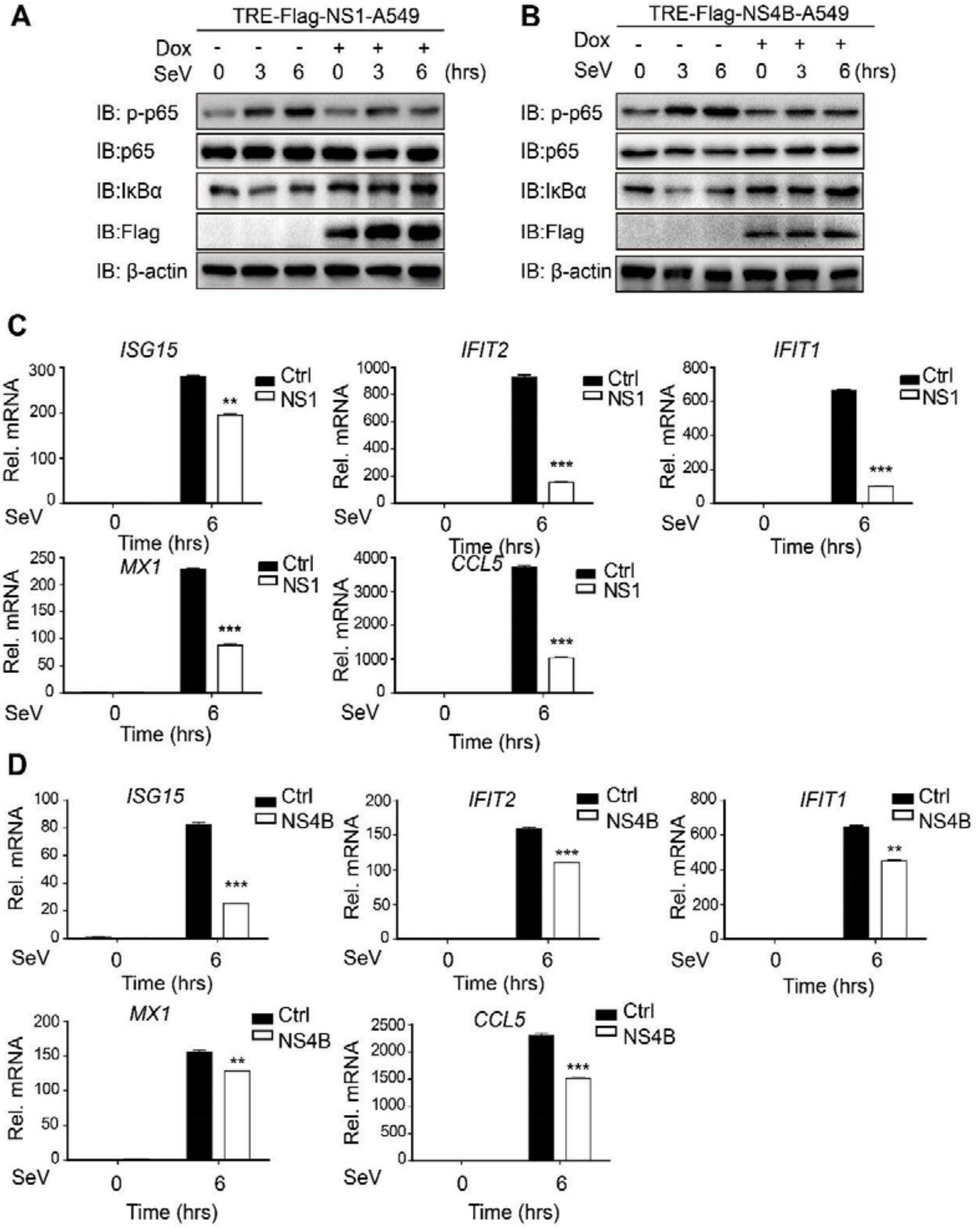

Supplementary Figure S2. ZIKV NS1 and NS4B inhibit IFNβ and ISGs expression.

(**A-B**) Immunoassay of extracts of NS1- or NS4B- inducible A549 cells, which were treated with doxycycline (Dox) (200 ng/mL) for 24 hrs, followed by SeV (MOI=0.1) for the indicated time points. (**C**) qRT-PCR analysis of *ISG15*, *IFIT2*, *IFIT1*, *MX1* and *CCL5* mRNA in NS1-inducible A549 cells after SeV infection for 4 hrs. (**D**) qRT-PCR analysis of *ISG15*, *IFIT2*, *IFIT1*, *MX1* and *CCL5* mRNA in NS4B-inducible A549 cells after SeV infection for 4 hrs. Data in **C** and **D** are expressed as means ± SD of at least three independent experiments. **p<0.01, *** p<0.001.

Figure S3

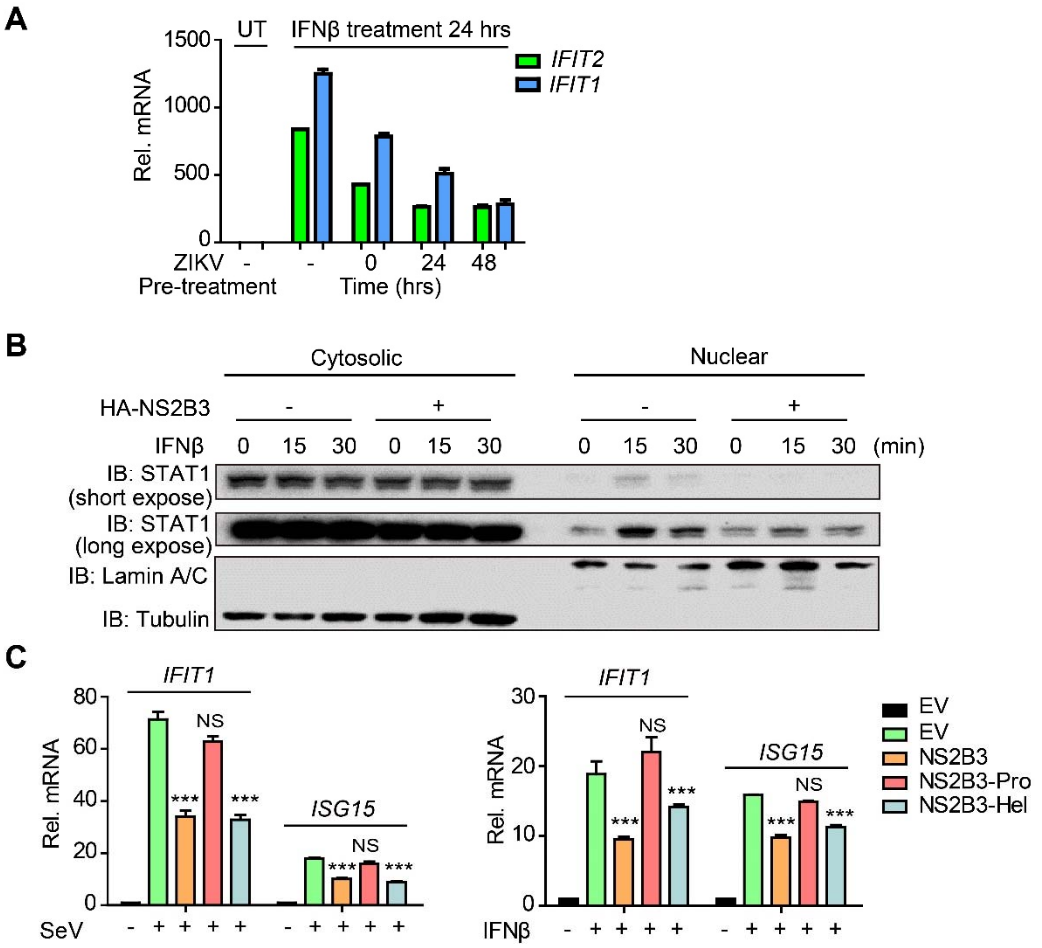

Supplementary Figure S3. ZIKV and its NS2B3 inhibit STAT1 nuclear translocation and ISGs expression.

(**A**) qRT-PCR analysis of A549 cells pre-infected by ZIKV, followed by IFNβ treatment. UT, untreated. (**B**) Immunoassay of cytoplasmic and nuclear extracts of 293T cells transfected with empty vector (EV) or HA-NS2B3 followed by IFNβ treatment for the indicated time points. Purity of cytoplasmic and nuclear fraction is depicted by tubulin and lamin A/C. (**C**) qRT-PCR analysis of *IFIT1* and *ISG15* mRNA in 293T cells transfected with NS2B3, NS2B3 protease domain or NS2B3 helicase domain after SeV infection or IFNβ treatment. Data in **A** and **C** are expressed as means ± SD of at least three independent experiments. *** $p<0.001$.

Figure S4

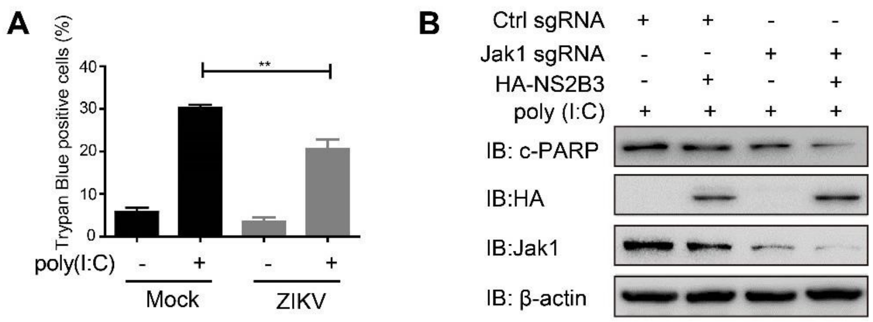

Supplementary Figure S4. ZIKV inhibits cytoplasmic poly (I:C)-induced cell death. (**A**) HT1080 cells pre-infected with ZIKV were treated by cytoplasmic poly (I:C). Trypan blue positive dead cells were then counted. (**B**) Immunoassay of extracts of indicated HT-1080 cells transfected with HA-NS2B3 and cytoplasmic poly(I:C). Data in **A** are expressed as means ± SD of at least three independent experiments. **$p<0.01$.

Figure S5

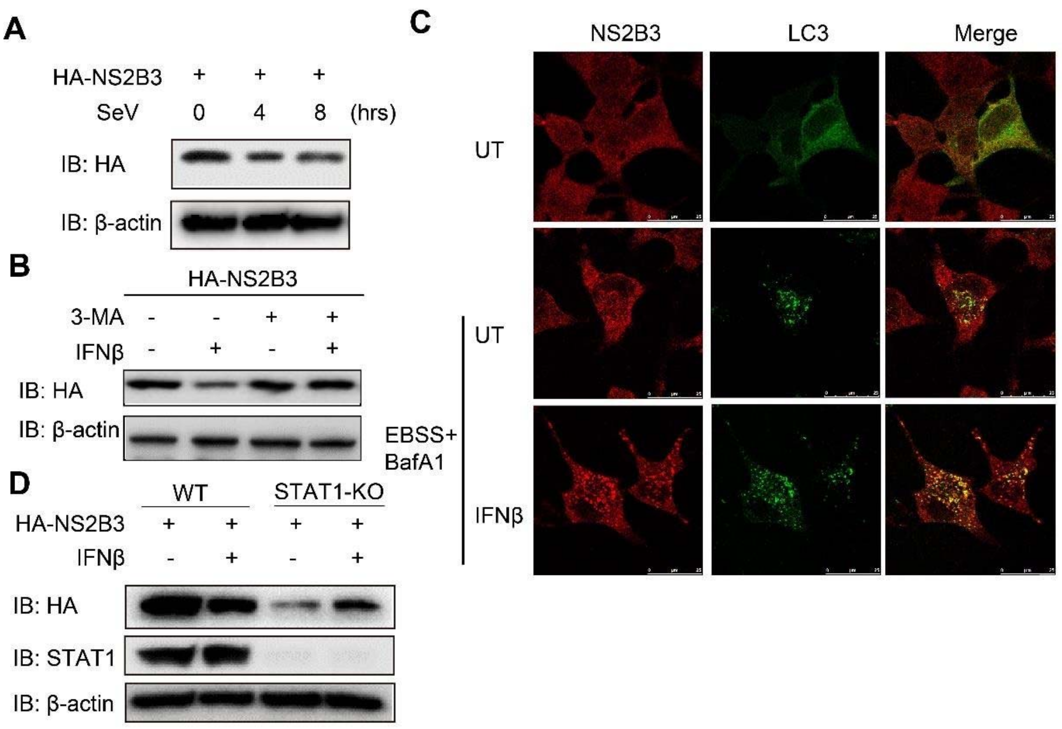

Supplementary Figure S5. IFNβ promotes the autophagic degradation of NS2B3. (**A**) Immunoassay of extracts of 293T cells transfected with HA-NS2B3 followed by SeV infection for the indicated time points. (**B**) Immunoassay of extracts of 293T cells treated with or without IFNβ after transfected with HA-NS2B3 and treated with DMSO or 3-MA (20 mM) for 4 hrs. (**C**) Confocal microscopy analysis of HeLa cells transfected with NS2B3 and LC3 treated with IFNβ, EBSS and BafA1 or not. UT: untreated. (**D**) Immunoassay of extracts of WT, and STAT1knockout (KO) 293T cells transfected with HA-NS2B3, followed by IFNβ treatment.
